# Efficacy and Safety of Acupuncture against Chemotherapy-Induced Peripheral Neuropathy: A Systematic Review and Meta-Analysis

**DOI:** 10.1155/2020/8875433

**Published:** 2020-11-09

**Authors:** Yuxia Jin, Yunxia Wang, Jiayuan Zhang, Xiao Xiao, Qi Zhang

**Affiliations:** Chengdu University of Traditional Chinese Medicine, No. 37 Shierqiao Road, Jinniu District, Chengdu, Sichuan 610075, China

## Abstract

**Objective:**

To determine the effectiveness and safety of acupuncture for chemotherapy-induced peripheral neuropathy. The review has been registered on the “PROSPERO” website; the registration number is CRD42020151654.

**Methods:**

A comprehensive literature search was performed on 7 electronic databases from the time of inception to March 2020. RCTs studies on acupuncture for CIPN compared with medication or sham acupuncture were included. Statistical analysis was carried out using RevMan 5.3.

**Results:**

In total, 19 RCTs covering 1174 patients were enrolled. The results showed that acupuncture significantly increased the effective rate of CIPN compared with medicine and sham acupuncture. And acupuncture had a good effect on the recovery of nerve conduction velocity and improving pain. Among the acupoints involved in the treatment of CIPN, LI4, LI11, ST36, EX10 (Bafeng), and EX-UE 9 (Baxie) were the most commonly used.

**Conclusion:**

The use of acupuncture in the management of CIPN is safe and effective. The most used acupoints for CIPN are LI4, LI11, ST36, EX10 (Bafeng), and EX-UE 9 (Baxie).

## 1. Introduction

Chemotherapy-induced peripheral neuropathy (CIPN) has a severe dose-limiting side effect for cancer patients treated with taxanes, vinca alkaloids, platinum drugs, and proteasome inhibitor bortezomib [1]. It is caused by damage or impairment in the dorsal root ganglia (DRG); that is to say, most of the neuropathy is sensory neuropathy, less involving motor and autonomic nerves [2]. It can manifest as numbness, tingling, and burning pain and mostly present in a stocking-and-glove distribution which reduces patients' quality of life and leads to patients' chemotherapy dose reductions or treatment discontinuation [3–5]. A recent meta-analysis [6] demonstrated a CIPN prevalence of 68% in the first month after chemotherapy and 30% at 6 months or more. Every third patient (37%) with neuropathic symptoms ranked them as the most troublesome symptom [7].

Currently, the clinical prevention and treatment of CIPN has no recognized specific means. The effectiveness of drugs used to treat other types of neuropathy may not extend to CIPN, such as gabapentin [8], tricyclic antidepressant amitriptyline [9], and opioids [10]. The only successful CIPN trial [11] shows that the antidepressant duloxetine at 30 mg daily for 1 week followed by 60 mg daily for 4 more weeks produced a significant reduction in BPI-SF average pain compared with the placebo arm (mean change score, 1.06 vs. 0.34; *P*=0.03). But it also has grades 2 to 3 adverse events (mainly fatigue and nausea), and 12 patients discontinued treatment due to toxicity. Therefore, acupuncture, a common adjuvant therapy, has been used encouragingly to treat peripheral neuropathy associated with diabetes [12] and HIV [13] and has attracted interest. Acupuncture is widely used in cancer-related diseases such as hot flashes [14],cancer-related fatigue [15],cancer-related insomnia [16],and leukopenia [17], and it can also reduce pain in arthritis [18–20],sciatica [21, 22], and gout [23]. Although several studies [24–42] have suggested that acupuncture was effective for treating CIPN, few systematic reviews have been published. We performed a systematic review and meta-analysis to assess the strength of the current evidence to support the efficacy and safety of acupuncture and electroacupuncture to treat CIPN, which might be a complementary therapy for CIPN.

## 2. Methods

### 2.1. Eligibility Criteria

The included studies met the following eligibility criteria. (1) Studies were designed up to randomized controlled trial (RCT) standards. (2) The patients who had malignant tumor received chemotherapy with peripheral neurotoxic drugs and had symptoms related to peripheral nerve injury. (3) The control group was treated with conventional methods, such as the use of neurotransmitters and antiepileptic drugs, or sham acupuncture.

The exclusion criteria were as follows: (1) Studies were nonrandomized controlled trials. (2) The experimental group was treated with pharmacoacupuncture, auricular acupuncture, laser acupuncture, or other uncommon forms of acupuncture. (3) Studies described interventions combined with other TCM therapies such as Chinese herbal medicine, acupoint injection, or herbal extracts. (4) Patients with original nervous system disease or peripheral neuropathy caused by other diseases.

### 2.2. Data Sources and Search Strategy

The following seven electronic databases were searched to identify eligible trials published from inception to March 15, 2020: Cochrane Central Register of Controlled Trials, Cochrane Database of Systematic Reviews, PubMed, EMBASE, Chinese Biomedical Literature Database, Chinese National Knowledge Infrastructure, and the Wanfang database. The 2 key concepts used in the search, peripheral neuropathy and acupuncture, included their synonyms. Two reviewers (JYX and ZJY) independently screened the titles and abstracts for eligibility and examined the full text of the articles. Any discrepancies were resolved by consensus or after consulting a third party (ZQ). The search keywords used were “Chemotherapy-induced peripheral neuropathy,” “Peripheral neuropathy,” “Neurotoxicity,” “Acupuncture,” “Acupoints,” “Needle,” as well as “random,” “clinical” and there was no restriction on language. More details have been provided on the “PROSPERO” website, and the registration number is CRD42020151654.

### 2.3. Data Extraction

Two reviewers (JYX and WYX) independently extracted data using an extraction sheet. The extracted data included general trial characteristics (authors and year); baseline patient and disease data (sample size, age, and gender); and interventions and outcomes. We contacted the authors via e-mail or telephone to acquire further information when the information in the literature was incomplete.

### 2.4. Quality Assessment and Data Analysis

Two reviewers (JYX and XX) independently assessed the methodological quality of the RCTs using the Cochrane Collaboration Risk of Bias tool. The risk of bias was assessed according to the Cochrane Handbook, which consists of six items: random sequence generation (i.e., selection bias), allocation concealment (i.e., selection bias), blinding of participants and personnel (i.e., performance bias), blinding of outcomes assessment (i.e., detection bias), incomplete outcomes data (i.e., attrition bias), selective reporting (i.e., reporting bias), and other biases. Discrepancies in this interpretation were resolved by consensus or after discussion with a third party (ZQ).

The data were analyzed using Review Manager 5.3 software (Cochrane Collaboration, Oxford, UK). The results are presented as the risk ratios (RR) or mean diﬀerences (MD) with the 95% confidence interval (95% CI). I^2^ statistics were used to assess heterogeneity.

## 3. Results

**Figure 1:**
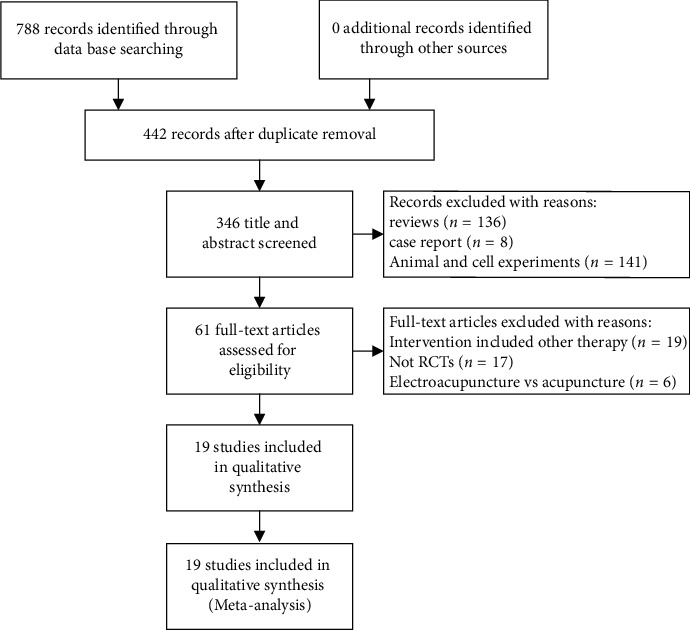
Flowchart of study selection.

The search results are displayed in Figure 1. 788 publications were identified in the literature search, and 442 duplicated citations were excluded. After screening the titles and abstracts, 285 irrelevant publications were excluded. After reading the full text of the remaining studies, 41 records were excluded. Finally, 19 RCTs [24–42] met the eligibility criteria and were included in the systematic review.

### 3.1. Characteristics of Included Trials

19 RCTs met the inclusion criteria: 1 study was conducted in Germany [42], 2 in the United States [36, 41], 1 in Brazil [40], and the other 16 studies in China. In total, 1174 patients (588 in the acupuncture or electroacupuncture group, 586 in the control group) participated. The characteristics of the included studies are shown in Table 1.

### 3.2. Risk of Bias and Methodological Quality

**Figure 2:**
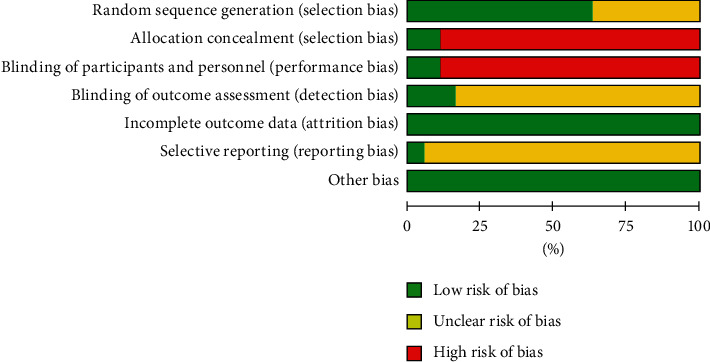
Risk of bias graph.

The assessment of risk of bias is summarized in Figure 2. All studies were described as “randomized” and 10 studies [24, 27, 28, 31, 33, 34, 36, 38, 40, 42] described appropriate methods for sequence generation. The remaining RCTs did not report specific random methods. One study [36] conducted allocation concealment.

### 3.3. Outcomes

#### 3.3.1. Total Effective Rate

Use the nimodipine scoring method to determine whether it is markedly effective, effective, or ineffective: markedly effective—the assessment of peripheral neurotoxicity is reduced by 2 levels or higher; effective—reduced by 1 level; and ineffective—the peripheral neurotoxicity is not reduced or increased; total effective rate = markedly effective rate + effective rate. Although all studies used the scale to evaluate patients, the results of some scales are expressed in the “I/II/III;/IV;” levels, which is not convenient to compare the scores as a continuous variable. Therefore, the change of patients' grades is used to show whether the treatment is effective, and by comparing the effective rate, the improvement degree of the disease is reflected.

**Figure 3:**
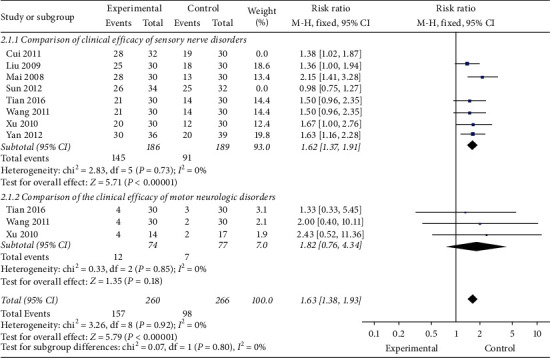
Total effective rate.

Only 8 studies [25, 26, 28, 29, 30, 33–35] (*n* = 375) were evaluated in this way. These studies showed no heterogeneity; thus, a fixed-effects model was used for statistical analysis. As shown in Figure 3, there is a significant difference between the experimental groups and the control groups in total effective rate (RR, 1.63; 95% CI, 1.38 to 1.93; *P* < 0.00001; and *I*^2^ = 0%). In the subgroup analysis, we found that acupuncture improved the sensory function significantly (RR, 1.62; 95% CI, 1.37 to 1.91; *P* < 0.00001; and *I*^2^ = 0%), while the improvement of motor function was not obvious (RR, 1.82; 95% CI, 0.76 to 4.34; *P*=0.18; and *I*^2^ = 0%).

**Figure 4:**
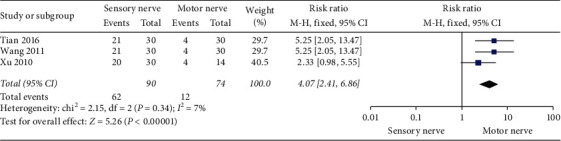
Sensory nerve vs. motor nerve.

As shown in Figure 4, 3 studies [28, 33, 34] compared the improvement of sensory and motor nerve disorders. The results showed that the recovery of sensory function was better than that of motor function (RR, 4.07; 95% CI, 2.41 to 6.86; *P* < 0.00001; and *I*^2^ = 7%).

#### 3.3.2. Nerve Conduction Velocity (NCV)

**Figure 5:**
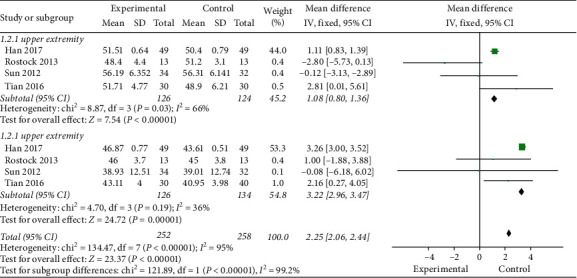
SCV.

*(1) Sensory Nerve Conduction Velocity (SCV)*. As shown in Figure 5, 4 studies [34, 35, 37, 42] (*n* = 250) were included. A significant improvement in SCV in the experimental group (MD = 2.25, 95% CI 2.06 to 2.44; and *P* < 0.00001), with high heterogeneity (*I*^2^ = 95%). And acupuncture is effective for both upper (MD = 1.08, 95% CI 0.80 to 1.36; and *P* < 0.00001) and lower (MD = 3.22, 95% CI 2.96 to 3.47; and*P* < 0.00001) extremities. Because of the high heterogeneity, sensitivity analysis was performed on the results. The results showed that the heterogeneity decreased significantly （*I*^2^ = 51%）when Han's results was removed.

**Figure 6:**
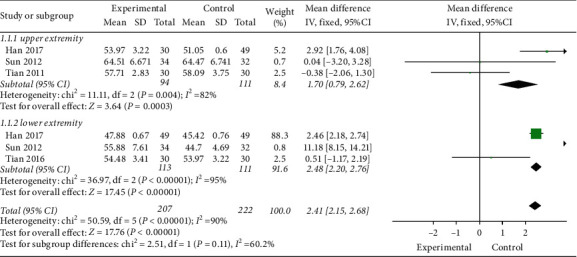
MCV.

*(2) Motor Nerve Conduction Velocity (MCV)*. As shown in Figure 6, 3 studies [34, 35, 37] (*n* = 205) were included. A significant improvement in MCV in the experimental group (MD = 2.41, 95% CI 2.15 to 2.68; and *P* < 0.00001), with high heterogeneity (I^2^ = 90%). And acupuncture is effective for both upper (MD = 1.70, 95% CI 0.79 to 2.62; and *P*=0.0003) and lower (MD = 2.48, 95% CI 2.20 to 2.76; and *P* < 0.00001) extremities.

**Figure 7:**
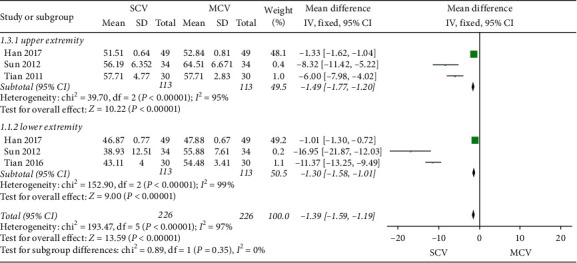
SCV vs. MCV.

*(3) SCV vs. MCV*. As shown in Figure 7, 3 studies [34, 35, 37] (*n* = 226) compared the SCV and MCV. The results showed that the recovery of SCV was worse than MCV (MD = −1.39, 95% CI −1.59 to −1.19; and *P* < 0.00001) with high heterogeneity (*I*^2^ = 97%).

#### 3.3.3. FACT-NTX Scores

**Figure 8:**
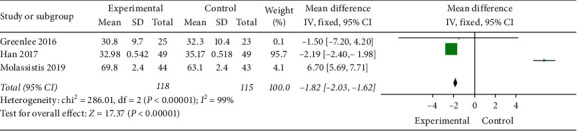
FACT-NTX scores.

As shown in Figure 8, 3 studies [36–38] (*n* = 233) compared the FACT-NTX scores. A significant reduction in FACT-NTX scores in the experimental group (MD = −1.82, 95% CI −2.03 to −1.62; and *P* < 0.00001), with high heterogeneity (*I*^2^ = 99%).

#### 3.3.4. BPI Scores

**Figure 9:**
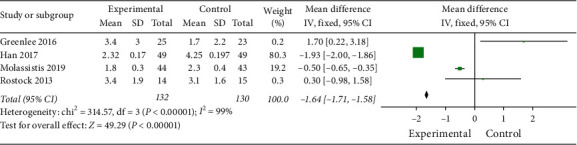
BPI scores.

As shown in Figure 9, 4 studies [36–38, 42] (*n* = 262) compared the BPI scores. A significant reduction in the experimental group (MD = −1.64, 95%  CI −1.71 to −1.58; and *P* < 0.00001), with high heterogeneity (*I*^2^ = 99%).

#### 3.3.5. Adverse Events

One trial [36] reported mild adverse events which were discomfort, minor swelling, and bruising after acupuncture needle withdrawal. Three trials reported [24, 33, 41] that no adverse events occurred. The remaining trials did not mention any adverse events.

## 4. Discussion

### 4.1. Analysis of Efficacy

This systematic review shows that the overall effectiveness of acupuncture treatment of CIPN is superior to that of neurotrophic drugs (Figure 3). Meanwhile, the recovery of sensory function is significantly better than motor function (Figure 4). It is interesting to see that many non-CIPN symptoms [26, 28, 31, 38] caused by chemotherapy, like nausea, appetite change, and drowsiness, had also improved,suggesting that acupuncture for CIPN can impact on a wider range of symptoms. However, on the other hand, the results [26, 40] show that acupuncture for mild and moderate limb numbness, pain, and other sensory disorders is significantly effective, while the curative effect for severe CIPN patients was poor. And measurable results can only be found after a longer period of treatment (up to 10 weeks) [43]. While the mechanism of action of acupuncture is not completely understood,several animal studies [44, 45] have reported that acupuncture can stimulate C nerve fibers which can be damaged from chemotherapy and enhance spinal/central GABA receptors, serotoninergic, and adrenergic neurotransmission [46]. Other hypotheses including “de qi” sensation,the principal of acupuncture,can increase blood flow in the capillaries,release local opioid peptides, stimulate specific areas of the brain [47], and participate in signal integration of central nervous system [48].

### 4.2. Analysis of NCV

The result shows that acupuncture has a good effect on the recovery of nerve conduction velocity (Figures 5 and 6), and the recovery of motor nerve function is better than that of sensory nerve function (Figure 7), but this is contrary to the results of the above questionnaire survey (Figure 4). This may be related to the fact that the motor nerve recovery is covered up by the decrease of myodynamia due to reduced exercise. The evidence shows that long-term aerobic exercise training can prevent the onset or modify the natural history of diabetic peripheral neuropathy [49]. This conclusion has also been confirmed in an animal experiment [50]. More favorable outcomes of peripheral neuropathy and psychosocial functioning are also reported in ovarian cancer survivors who were meeting physical activity guidelines [51]. These articles suggest that moderate exercise may play an important role in the treatment of CIPN. On the other hand, questionnaires and self-reports are the preferred measure of CIPN [52], but they can be highly susceptible to subjective factors. Significant statistical difference is found in the change of mean values of the NCI-CTCAE Scale in Alessandro et al.'s experiment [40], and some literatures [53] think that the assessment modalities for neuropathy may overestimate the presence of motor neuropathy and misdiagnose CIPN. So, current scales may not be appropriate to measure CIPN in a valid way. More objective outcome measurements and a combination of scales for the standardization of CIPN are advisable.

### 4.3. Analysis of FACT-NTX Score

We can see the positive effect of acupuncture in the analysis of FACT-NTX score (Figure 8), but the results are highly heterogeneous. It may be due to the fact that FACT-NTX score is a subjective index, and results are easily affected by artificial errors. The different therapeutic effects caused by different acupoints may also be the cause of differences. We found that the acupoints in Yangming Meridian are generally used for the treatment of CIPN because “treatment for flaccidity aims at Yangming meridian” is a therapeutic principle of acupuncture in traditional Chinese medicine, which means that the acupoints on Yangming Meridian like LI4, LI11, and ST36 are often used in the treatment of muscle atrophy and peripheral neuropathy, such as CIPN, diabetic peripheral neuropathy, stroke, and myasthenia gravis. Yangming Meridian is also closely related to the gastrointestinal system, so it can explain that in the treatment of CIPN, gastrointestinal related symptoms like nausea, loss of appetite, and fatigue are also improved. Another characteristic of the treatment of CIPN is using local points on the extremes like EX10 (Bafeng) and EX-UE 9 (Baxie) to stimulate the peripheral nerves and improve the local symptoms of the limbs.

### 4.4. Analysis of BPI Score

Although the result shows that acupuncture has a certain effect on improving pain, the heterogeneity of the results is high. In Rostock et al.'s study [42], there is no therapeutic advantage of electroacupuncture over an orally administered placebo control. They attribute it to a floor effect that their study design is based on patients with more severe CIPN symptoms and higher pain scores,so there are no large margins for many patients to improve considerably. Alessandro et al. and Yan et al.'s studies [26, 40] also show that the benefits of acupuncture for pain are moderate and appear to be more significant for patients with recent neuropathy symptoms. In Greenlee et al.'s study [36],the pain symptoms become worse after the postintervention in the electroacupuncture arm,which is similarly reported in Chen et al.'s RCT [54] of electroacupuncture in reducing pancreatic cancer pain. The mechanism is not clear, but we found that both Rostock et al. and Greenlee et al.'s studies use electroacupuncture. The strong stimulation of electroacupuncture may aggravate the pain. Therefore, electroacupuncture may not be as effective and safe as traditional acupuncture in CIPN.

### 4.5. Limitations of Study

We only searched the studies from the English and Chinese databases, so some studies may be omitted, which may lead to the sampling bias. The sample size included in the study is small and because there is no generally agreed-upon placebo control, the control group is inconsistent, which may affect the accuracy of the effectiveness evaluation. Lack of blinding of both subjects and investigators is a common methodological flaw of the selected studies, which means positive expectation may amplify acupuncture's effect. Therefore, the findings of this study should be interpreted cautiously.

## 5. Conclusion

In conclusion, our analysis indicates that the use of acupuncture in the management of CIPN is safe and effective. The most used acupoints for CIPN are LI4, LI11, ST36, EX10 (Bafeng), and EX-UE 9 (Baxie). However, considering the methodological limitation of the studies, caution needs to be taken in applying the conclusions of this review. The existing studies lack long-term observation. Furthermore, none of the included studies compared acupuncture with duloxetine [3], a drug which is currently the most effective treatment in CIPN. Thus, we would recommend that future studies use this medication as a comparator and observe the long-term curative effect of acupuncture.

## Figures and Tables

**Table 1:** Characteristics of the included RCTs.

Study ID	Sample size (T/C)	Age (year)	Sex (M/F)	Intervention		Outcome measure	Acupoints
				Treatment group	Control group		
Hou, 2011	40 (20/20)	T: 59.20 ± 9.80	T: 11/9	Acupuncture	Cobaltine adenosine	NCI-CTC	LI11, PC6, LI4, ST36, SP6, and GB34
		C: 62.95 ± 10.85	C: 8/12				
Mai, 2008	60 (30/30)	T: 50.35	T: 20/10	Acupuncture	VB_1_ + VB_12_	PNQ	GB20, TE5, ST36, BL40, GB34, ST40, SP6, LR3, and SP10
		C: 51.12	C: 21/9				
Yan, 2012	75 (36/39)	T: 52.5 ± 5.63	T: 20/16	Acupuncture	VB_1_ + VB_12_	PNQ	LI4, LI11, EX-UE9, LR3, ST36, SP10, and EX-LE10
		C: 51.3 ± 6.17	C: 28/11				
Xu, 2010	64 (32/32)	T: 38∼77	T: 17/15	Acupuncture	VB_1_ + VB_12_	PNQ	LI4, LR3, ST36, CV6, LI11, SP3, EX-UE9, and EX-LE10
		C: 41∼83	C: 16/16				
Liu, 2009	60 (30/30)	T: 64.8	T: 16/14	Acupuncture	Cobaltine adenosine	Levi	LI11, LI4, ST36, SP6, GB34, KI16, CV6, and CV4
		C: 62.6	C: 17/13				
Cui, 2011	62 (32/30)	T: 59.6	T: 19/13	Acupuncture	Cobaltine adenosine	Levi	LI11, TE5, SI3, LI4, SJ3, SP9, ST36, GB34, SP6, GB39, and KI6
		C: 58.8	C: 20/10				
Chen, 2018	80 (40/40)	T: 49.02 ± 8.63	T: 24/16	Acupuncture	Mecobalamin	RECIST	ST36, CV6, SP3, LI4, LI11, LR3, EX-UE9, and EX-LE10
		C: 48.75 ± 8.67	C: 25/15				
Wang, 2011	60 (30/30)	T: 63.7 ± 9.8	T: 16/14	Acupuncture	Cobaltine adenosine	PNQ	LI4, LI10, LI1, ST32, ST36, ST40, GB30, GB31, GB34, EX-UE9, and EX-LE10
		C: 61.6 ± 8.9	C: 17/13				
Tian, 2016	60 (30/30)	T: 60.9 ± 10.7	T: 18/12	Acupuncture	Cobaltine adenosine	PNQ, NCV, and QOL	LI10, LI11, LI4, TE5, EX-UE9, GB34, ST40, SP6, ST36, GB40, LR3, EX-LE10, CV4, CV6, and GV20
		C: 62.7 ± 10.4	C: 17/13				
Han, 2017	98 (49/49)	—	T: 27/22	Acupuncture	Cobaltine adenosine	VAS, NCV	LR3, ST43, GB4, SP6, ST36, SP10, ST25, GV14, GV12, GV11, GV9, and BL13 (BL17, BL58)
		—	C: 29/20			FACT/GOG-NTX	
Sun, 2012	66 (34/32)	T: 64	T:24/10	Electroacupuncture	Reduced glutathione	Levi, NCV	ST36, SP10, CV4, LI11, LI4, SJ10, TE4, LI5, SI4, EX-UE9, SI3, ST35, ST34, SP9, SP6, ST41, KI3, BL60, BL62, KI6, LR3, and EX-LE10
		C: 61	C: 22/10				
Tian, 2011	76 (38/38)	T: 54.9	T: 24/14	Acupuncture	Neurotropin	Levi, KPS	LI4, TE5, LI5, LI10, LI11, LR3, ST36, GB34, CV6, ST40
		C:52.7	C: 20/18				
Zhang, 2018	30 (15/15)	T: 52.46 ± 10.06	T: 10/5	Electroacupuncture	VB_1_ + VB_12_	Levi, KPS	LI11, LI10, LI4, ST36, ST40, ST44
		C: 56.80 ± 8.06	C: 8/7				
Greenlee, 2016	63 (31/32)	T:51.8	—	Electroacupuncture	Sham acupuncture	BPI-SF, FACT-NTX	GB34, ST36, LI4, LI10, L3, L5, C5, and C7
		C:48.3	—				
Molassistis, 2019	87 (44/43)	—	T: 9/35	Acupuncture	VB_6_ + VB_12_	BPI, FACT-NTX	If upper limbs were involved, we used LI4, LI11, PC7, TE5, and/or Ex-UE9; if lower limbs were affected (most common), we used SP6, ST36, LV3, ST41, and/or Ex-LE10
		—	C: 15/28			Neuropathy score	
Rostock, 2013	29 (14/15)	T:49.9 ± 9.6	T: 4/10	Electroacupuncture	VB_6_ + VB_1_	NCI-CTC, NRS	LV3, SP9, GB41, GB34, LI4 , LI11 , SI3 , and HT3
		C:56.3 ± 11.1	C: 5/10			Neuropathy score, NCV, and QLQ-C30	
Wu, 2018	40 (20/20)	T:57.10	T: 13/7	Acupuncture	Mecobalamin	Levi	LI11, PC6, LI4, ST36, SP6, and SP10
		C:57.63	C: 14/5				
Lu, 2019	40 (20/20)	T:54.0	T: 0/20	Acupuncture	Usual care	PNQ, FACT-NTX, BPI-SF, and EORTC QLQ-C30	Yin Tang, LI11, TW5, Ex-UE9, SP3, ST36, SP6, K3, and LR3
		C:53.5	C: 0/20				
Alessandro, 2019	21 (12/9)	—	—	Acupuncture	Usual care	NCI-CTC, EORTC QLQ-C30, FIM, and VAS	Yuan points of the hands and feet (LR3, SP3, KI3, HT7, PC7, and LU9) plus SP9 and wrist-ankle technique (areas 1–3)
		—	—				

Abbreviations: T: Treatment Group; C: Control Group; NCI-CTC: National Cancer Institute Common Toxicity Criteria; PNQ: Patient Neurotoxicity Questionnaire; RECIST: Response Evaluation Criteria in Solid Tumors; NCV: Nerve Conduction Velocity; QOL: Quality of Life; VAS: Visual Analogue Scale; KPS: Karnofsky; MDASI: MD Anderson Symptom Inventory: BPI-SF, Brief Pain Inventory-short form; EORTC QLQ-C30, European Organisation for Research and Treatment of Cancer Quality-of-Life Questionnaire Core 30; FACT-NTX, Functional Assessment of Cancer Therapy-Neurotoxicity subscale; FIM: Functional Independence Measure.

## Data Availability

All the data were taken from the published studies.
